# Study on the differences in peripheral fatigue responses elicitation effects of bench press training with different loads and tempos based on electromyography and motion sensors

**DOI:** 10.3389/fphys.2025.1661217

**Published:** 2025-09-23

**Authors:** Bin Yan, Zhicong Li, Chunwei Li, Chongyang Han, Siqi Yao, DongDong Ma, Wenzhong Zhang

**Affiliations:** ^1^ Academic Affairs Office, Henan Sport College, Zhengzhou, China; ^2^ School of Physical Fitness and Arts, Wuhan Sport University, Wuhan, China; ^3^ Department of Physical Education, Tianjin Medical University, Tianjin, China

**Keywords:** exercise tempo, bench press, neuromuscular fatigue, resistance training, blood lactate

## Abstract

**Objective:**

This study aimed to investigate the effects of bench press training with different loads (60% 1RM vs. 80% 1RM) and tempos (maximal velocity X/0/X/0 vs. medium tempo 2/0/2/0) on peripheral fatigue responses in bodybuilders, assessing the specific roles of neuromuscular activation, metabolic stress, and kinetic performance.

**Methods:**

Ten experienced male bodybuilders performed four training protocols to exhaustion in a randomized crossover design. Electromyography (EMG) was used to record muscle activation (normalized as %MVIC) and spectral shifts (Median Frequency - MDF) from the pectoralis major, anterior deltoid, and triceps brachii muscles. Biochemical assessment involved measuring blood lactate concentrations pre- and post-exercise to quantify metabolic stress. Motion sensors (Vmaxpro) were employed to capture barbell kinematics—including mean velocity (MV), peak velocity (PV), mean power (MP), peak power (PP), and time under tension (TUT)—providing direct measures of neuromuscular performance and fatigue-related velocity loss.

**Results:**

A significant interaction between load and tempo was found for all fatigue markers (p < 0.05). The combination of high load and fast tempo (80% 1RM, X/0/X/0) induced the most pronounced peripheral fatigue, evidenced by the highest muscle activation (%MVIC) and blood lactate levels, coupled with the greatest declines in MDF (indicating neuromuscular fatigue), velocity, and power output.

**Conclusion:**

The interaction of load and tempo critically determines the pattern and magnitude of acute peripheral fatigue. High-load fast-tempo training elicits multifaceted fatigue across neuromuscular, metabolic, and performance domains, whereas a high-load medium-tempo protocol results in less fatigue despite longer TUT. These findings provide a scientific basis for precise fatigue management in resistance programming.

## 1 Introduction

In the realm of bodybuilding competition, an athlete’s stage performance is highly dependent on the precise control of muscle hypertrophy, definition, and symmetry (Alves et al., 2020). As the core training modality, resistance training plays a crucial role in achieving these goals. Among its variables, exercise load and tempo, as quantifiable key parameters, profoundly influence the intensity of muscle stimulation, the rate of metabolic stress accumulation, and the adaptive remodeling of the neuromuscular system (Wilk et al., 2020a). From a physiological perspective, medium-load (60% 1RM) training induces repeated muscle fiber contractions through higher repetition counts, which effectively stimulates satellite cell proliferation and muscle protein synthesis, making it particularly advantageous for promoting muscle hypertrophy and enhancing endurance (Enoka, 2021). In contrast, high-load (80% 1RM) training recruits more fast-twitch muscle fibers and activates the phosphagen metabolic system, thereby effectively increasing muscle strength and neuromuscular recruitment efficiency (Jenkins et al., 2016). Exercise tempo exerts a more complex regulatory effect on muscle fatigue. For instance, a slower tempo extends the muscle’s time under tension (TUT), which can exacerbate the micro-damage from eccentric contractions and the accumulation of metabolic byproducts, while a maximum velocity tempo challenges the neuromuscular system’s ability to generate force rapidly (Wilk et al., 2020b).

In the context of bodybuilding training, muscle hypertrophy is indeed influenced by a combination of factors, including training stimulus, fatigue accumulation, nutritional intake, and the recovery process (Alves et al., 2020). This study, however, focuses on fatigue as its core variable because training load and exercise tempo are the most direct and quantifiable parameters in resistance training, and peripheral fatigue is the immediate, measurable physiological response to these variables. From a physiological perspective, peripheral fatigue is a core limiting factor for training intensity and duration (Río-Rodríguez et al., 2016); Therefore, understanding how to manage fatigue by regulating these variables is the fundamental prerequisite for optimizing any training program. Despite the established independent importance of these variables, a significant gap and controversy exist in the literature regarding their interactive effects on peripheral fatigue responses (Wilk et al., 2020a). Previous research has mostly focused on a single variable in isolation, which limits the accurate assessment of fatigue risks in compound training programs. For instance, while the work of Wei Lu’s team (Lu et al., 2023) found that fast-tempo training under high load can enhance instantaneous power output, it did not delve into the potential fatigue costs or provide dynamic tracking of the fatigue accumulation process. Similarly, Zachary A. Mang’s research linked maximum velocity tempos with higher blood lactate levels, but their study was conducted only at a fixed 60% 1RM load, making it difficult to generalize the conclusions to other training intensity scenarios (He et al., 2024). The limitations of these single-variable studies mean that our understanding of the synergistic effects of load and tempo is limited. This research gap leads to a series of pressing practical questions. For example, does training with a 60% 1RM load combined with a maximum velocity tempo accelerate fatigue accumulation due to insufficient muscle recovery time, despite maintaining higher power output? Conversely, can an 80% 1RM load combined with a medium tempo optimize fatigue management by balancing metabolic stress and peripheral fatigue responses while extending time under tension? Currently, no studies have quantitatively analyzed this synergistic effect. Answering these questions holds significant practical value for the scientific formulation of bodybuilding training programs. Therefore, this study, utilizing a two-factor crossover experimental design, aims to systematically investigate the differences in peripheral fatigue responses among four combinations of medium/high load and fast/medium tempo in bodybuilders. The goal is to provide a theoretical basis and methodological support for formulating personalized resistance training programs and achieving a dynamic balance between training effectiveness and fatigue management. This study proposes the following hypotheses: under the condition of controlling the total amount of training (to exhaustion), high load (80%1RM) induced compensatory recruitment of high-threshold motor units compared with moderate load (60%1RM), which was manifested as a significant increase in muscle activation, but produced more significant exercist-induced fatigue. These effects were manifested as lower median electromyographic frequency (MDF), higher blood lactate level, and greater velocity and power attenuation, which were further amplified by fast rhythm than medium rhythm. In addition, there was an interaction between load and rhythm. That is, the combination of 80%1RM and X/0/X/0 will produce a synergistic effect, and its index changes are more significant.

## 2 Materials and methods

### 2.1 Study design and participants

The study was conducted at the Physical Training Center of Physical Education. A randomized crossover design with paired self-controls was employed. Recruitment focused on students from the institution; and strict criteria were applied to the initial pool of candidates; resulting in a final study group of 10 eligible participants. Their basic information is listed in Table 1. To eliminate inherent subjective bias; the true purpose of the experiment was withheld until the conclusion was drawn; and participants were only informed that the study aimed to explore the effects of high-load or fast-tempo bench press training on exercise fatigue. The decision to select male participants was made to control for hormonal and physiological variations that might affect muscle activation and training outcomes; ensuring the consistency of physiological measurements.

**TABLE 1:** Basic information of participants (*n* = 10).

Age (years)	Height (cm)	Weight (kg)	Bench press 1RM (X/0/X/0) (kg)	Bench press 1RM (2/0/2/0) (kg)
24.20 ± 1.66	173.80 ± 2.60	77.90 ± 5.48	120.00 ± 16.88	111.50 ± 15.66

We conducted a risk assessment of physical activity, reviewed the physical activity history of each participant, and had them complete the Physical Activity Readiness Questionnaire (PAR-Q+) to evaluate their health status and ensure the safety of the experimental protocol. The working environment was comprehensively assessed to meet safety standards. Before obtaining informed consent, participants were fully informed of the study’s purpose, methods, and potential risks. This study was approved by the Ethics Committee of Zhengzhou University (ZZUE20240922) and followed the principles of the Declaration of Helsinki. All participants provided their informed consent to participate in this study. The sample size for this study was determined using G*Power software (version 3.1). The parameters used in the analysis were as follows: an F-test for ANOVA (repeated measures, within factors), a desired power level of 0.8, and an expected effect size f = 0.56, which was based on prior studies (Zihan et al., 2024). Considering a potential 20% sample loss, the initial calculated sample size was seven subjects. Ultimately, a total of 10 participants were enrolled in the study. Through strict inclusion criteria, a final group of 10 qualified subjects was formed for the study, with their basic information detailed in Table 1.

Inclusion Criteria: (1) At least 2 years of resistance training experience. (2) Proficiency in performing the bench press, with the ability to complete a bench press at least 1.2 times their body weight. (3) No history of uncontrolled chronic diseases such as heart disease or hypertension.

Exclusion Criteria: (1) History of musculoskeletal injuries in the upper limbs or chest that could affect bench press performance. (2) Cervical or lumbar spine conditions that might be exacerbated by bench press activities. (3) Use of any substances or devices that could affect muscle strength or activity, such as weightlifting straps, wristbands, steroids, or stimulants.

### 2.2 Experimental design and process

**FIGURE 1:**
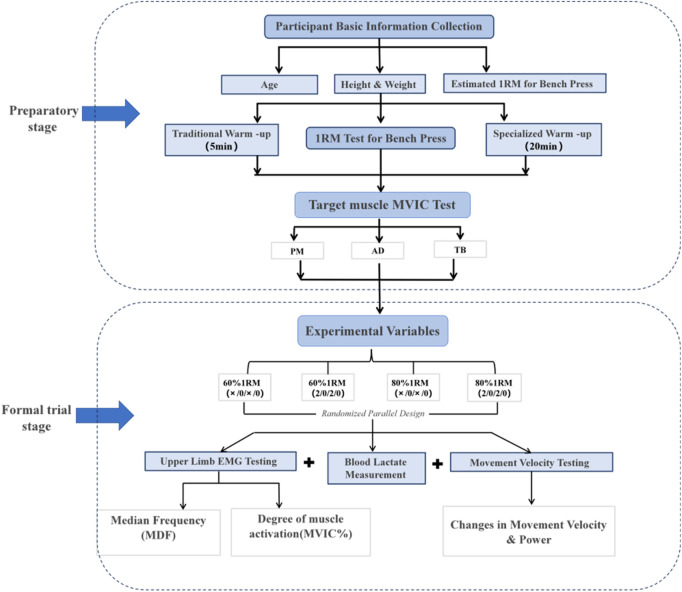
Experimental flowchart.

Ten bodybuilders with over 2 years of resistance training experience were selected for this study and randomly assigned to four different training modes using a lottery method. These training modes combined different training loads (medium intensity at 60% 1RM and high intensity at 80% 1RM) with different tempos (maximum velocity tempo at X/0/X/0 and medium tempo at 2/0/2/0). The maximum velocity tempo (X/0/X/0) refers to the bench press performed at a large velocity in eccentric and isometric phases. In contrast, the medium tempo (2/0/2/0) refers to a 2-s duration in both the eccentric and isometric phases of each bench press. Failure to maintain the 1-s pause or any visible bar bounce resulted in the repetition being discarded and repeated. To eliminate the potential interference of circadian rhythms, all experimental procedures were uniformly scheduled between 13:00 and 17:00. In the 24 h preceding the formal experiment, participants were required to strictly abstain from alcohol, caffeine-containing beverages (including coffee, tea, cola, energy drinks, and caffeine supplements), and high-intensity physical activities. During the first visit to the laboratory, participants’ primary task was to familiarize themselves with the entire experimental process, accurately master the execution standards of movements, and understand the criteria for each test. Under the guidance of the staff who would be responsible for the formal experimental testing, participants became thoroughly acquainted with the exercise testing procedures, received detailed explanations of safety precautions, and had their basic physiological data (such as height and weight) collected. During the formal experimental phase, a 72-h interval was set between each combination of tests to ensure physical recovery. Each participant was required to complete four sets of bench press training to exhaustion for each training combination, with a 5-min rest between sets. Exhaustion was defined as the inability to complete the concentric phase through the full range of motion, accompanied by visible form breakdown (bar path deviation, lifting of glutes/shoulders off bench) or failure to maintain required tempo for two consecutive repetitions (Krzykała et al., 2023). At three critical time points—before, during, and after training—the participants’ blood lactate concentration, EMG signal characteristics, and exercise performance indicators were precisely tested and meticulously recorded. The experimental process is shown in Figure 1.

### 2.3 Main test and observation indicators

#### 2.3.1 Bench press 1RM test

In this study, 1RM was determined 72 h before the first experimental session, participants were required to perform bench press training at two different tempos and movement trajectories during the 1RM test and the formal experiment (a metronome set at 60 beats per minute (BPM) to control the tempo of the participants: 1 beat = 1 s) training at maximum velocity tempo (X/0/X/0) in a non-explosive manner, with both the eccentric and concentric phases performed at maximum velocity tempo without pause; b) training at a medium tempo (2/0/2/0), with the eccentric phase lasting 2 s without pause at B point, and the concentric phase lasting 2 s without pause at C point, to ensure the consistency of movement timing as much as possible). The selection of 2/0/2/0 as the medium tempo was based on studies by Lu et al. (2023) and Wilk et al. (2020b). Tempo notation (eccentric/isometric/concentric/isometric) defines phase durations in seconds, where ‘X’ = maximal velocity, ‘0’ = no isometric pause, and ‘2’ = 2-s controlled duration. For the maximum velocity tempo (X/0/X/0), participants performed eccentric/concentric phases at maximal velocity with 0-s isometric pause between phases; additionally, a 0.5-s controlled hold at the bottom position (end of eccentric phase) was required to eliminate rebound effect (The 0.5-s bottom pause in the maximum velocity tempo was explicitly defined as an auxiliary control measure (not reflected in the “X/0/X/0” notation) to avoid conflation with the “0” isometric phase parameter). This “touch-pause-go” protocol removes the stretch-shortening cycle and ensures that the subsequent upward velocity reflects only voluntary concentric force production (Pallarés et al., 2014). Tempo was monitored with a metronome and Vmaxpro sensor; trials deviating >5% were discarded. A metronome was used to control the training tempo to ensure that the times of the eccentric and concentric phases met the protocol requirements. During the bench press training, the head, shoulders, and buttocks must remain in contact with the bench, the foot is divided into hip width, the sole of the foot is attached to the ground and stepped firmly, located under the knee or slightly in front, and the heel is not off the ground, the barbell must touch the chest during the descent, and the elbows must be fully extended during the push-up to achieve the complete standard of the bench press movement. The position of the hands on the barbell was fixed, equivalent to 150% of the individual’s biacromial distance. According to Michal Wilk’s team (Wilk et al., 2021a), only one repetition test was used to determine the 1RM (one-repetition maximum) in their study, without independent 1RM tests for other tempos. This may affect the comparison of related indicators under different tempos. Therefore, to address this limitation in the experiment, 1RM tests were conducted separately for different tempos. Participants tested the 1RM for two tempos (maximum velocity tempo X/0/X/0 and medium tempo 2/0/2/0) with a 72-h interval. The 1RM bench press test protocol is as follows: after familiarizing the subject with the procedure during warm-up, the initial load is set to 80% of their self-reported 1RM, with 4–9 kg increments for 3–5 repetitions per set, followed by 2-min rest intervals. Subsequent loads are increased by 10%–20% for 3–5 repetitions with another 2-min rest. After a third 10%–20% increment and 3–5 repetitions, 1RM attempts commence: 4–9 kg increments for successful lifts or 2–4 kg decrements for failures, repeated until the subject cannot complete the lift, with all 1RM values determined within 5 trials. Successful attempts require 2–4 min of rest before repeating the process, while failed attempts necessitate 2–4 min of rest and 5%–10% load reduction for re-testing, with load adjustments continuing until the subject achieves 1RM using proper bench press technique within 5 trials.

#### 2.3.2 Target muscle maximum voluntary isometric contraction (MVIC) test

**FIGURE 2:**
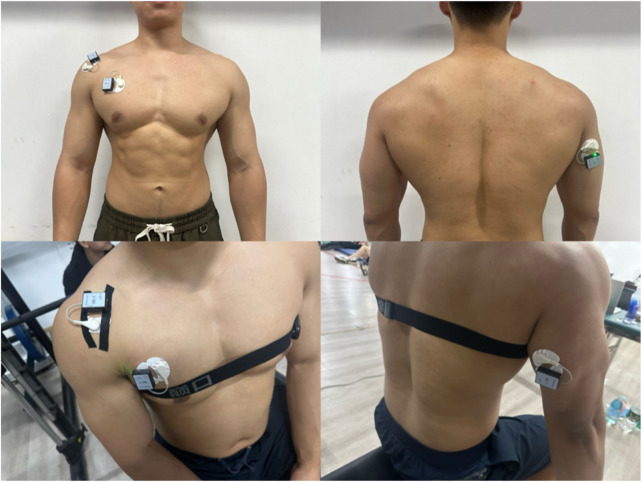
Electrode placement locations.

Prior to the formal test, a signal receiver was placed beside the participant and connected to the application software for a pretest. The pretest checked whether the EMG signal connection was complete and whether the signal was normal. Only after confirming that all preparations were in order was the MVIC test officially conducted. The MVIC test was completed before the formal bench press with different load and rhythm to determine the maximum muscle activation of each target muscle group, and then provide the necessary data for the subsequent normalization process to confirm the potential of the relative muscle activation (MVIC%) value of each muscle during exercise. This experiment used a 16-channel YW-Wireless wireless surface EMG system to measure and analyze the surface EMG signals of muscles (Model: YW-EMG16, YingWei Technology, Zhengzhou, China). Signals were collected using YW-EMG Analysis Software v2.0, and processed in MATLAB R2023a (MathWorks, United States) with the Signal Processing Toolbox and Statistics and Machine Learning Toolbox. Based on the biomechanical characteristics of the bench press movement, three muscles in the upper limb and synergistic muscle groups were included: pectoralis major, triceps brachii, and anterior deltoid. According to anatomical features, the electrodes were placed on the muscle bellies of these muscles. All EMG recordings were performed on the dominant limb (right for all participants) as determined by the Edinburgh Handedness Inventory (Ejavibulya et al., 2025) (Figure 2).

**FIGURE 3:**
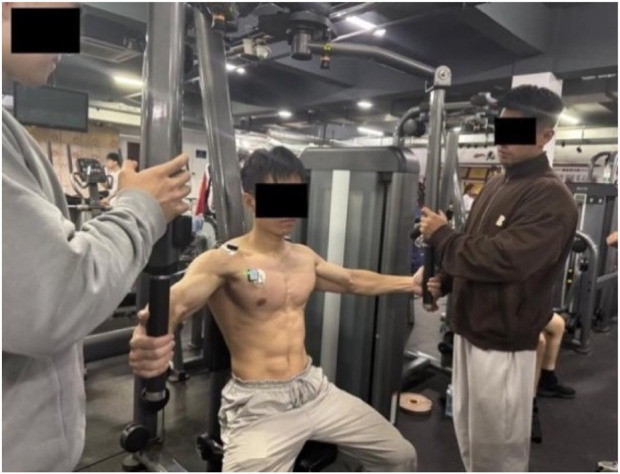
Pectoralis major muscle.

**FIGURE 4:**
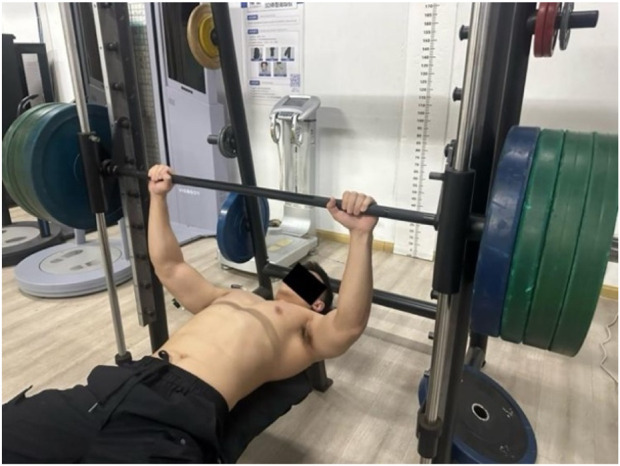
Anterior deltoid muscle.

**FIGURE 5:**
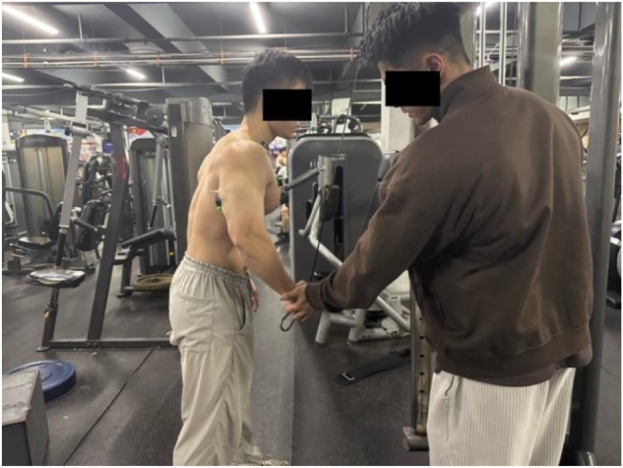
Triceps brachii muscle.

The integrated EMG values for each muscle during the MVIC test were collected according to the protocol described by He et al. (2024). The testing procedure is as follows (each muscle underwent two MVIC tests):

a. Pectoralis Major (Pectorales): Participants sat on the butterfly machine, keeping the back against the seat, chest straight, arms slightly flexed, and feet flat on the ground. The weight of the device was adjusted to close to the maximum weight the subject could bear. After hearing the password, the subject clamped the chest with the maximum force, held at the limit Angle for 3–5s, and the EMG data were collected, as shown in Figure 3. (Participants sat on the butterfly machine, which isolates pectoralis major contraction and ensures consistent isometric force production—critical for standardized MVIC measurement. This setup avoids the confounding effects of triceps or deltoid co-activation present in push-ups. For the bench press-specific movement, the bar was not fixed to replicate real-world training mechanics, allowing natural kinematics and muscle coordination (Dustin et al., 2015).)
b. Anterior Deltoid: Participants lay supine on the bench of a Smith machine, with their back tightly against the bench and hands gripping the barbell bar slightly wider than shoulder-width. After adjusting to the correct posture, the barbell was fixed on the Smith machine. Upon hearing the command, participants performed a bench press with maximum effort, holding the position for 3–5 s while EMG data were collected, as shown in Figure 4.
c. Triceps Brachii: The participant performed a single-arm extension using the cable attached to a power rack, with the upper arm held stationary against the body and a slight forward lean. The participant exerted maximum effort to straighten the forearm, while the tester applied a counterforce at the participant’s point of effort. The position was held for 3–5 s, during which EMG data were collected, as shown in Figure 5.

The following sections detail specific parameters and rationale during the analysis of EMG analysis, including filtering, rectification, smoothing, and normalization. Specifically, the raw surface EMG signals were acquired using a 16-channel YW-Wireless wireless acquisition system at a sampling rate of 2,000 Hz. Signals were processed offline using zero-phase forward and reverse Butterworth filters to avoid phase shifts. A multi-stage strategy is adopted in the filtering process: For noise interference in the original signal, a fifth-order Butterworth bandpass filter with a combination of 8 Hz high-pass and 450 Hz low-pass is actually used (instead of independent low-pass or high-pass filtering). The effective signal in the 8–450 Hz frequency band is retained bxy a zero-phase filtering algorithm (such as the filtfilt function), and the lower cut-off frequency of 8 Hz is selected. It can effectively remove low-frequency artifacts such as skin-electrode interface motion and baseline drift caused by breathing, sweat and other factors, which can contamize the signal and obscure the real muscle activity. An upper cutoff frequency of 450 Hz was chosen to eliminate high-frequency noise from external electromagnetic sources, such as power lines, laboratory equipment, while preserving the power of the vast majority of physiological signals, which are concentrated below this threshold. The bandpass filtering method ensures that the EMG signal is effectively isolated from irrelevant frequency components, thereby improving the accuracy and reliability of subsequent analysis. After bandpass filtering, a second-order zero-phase notch filter centered at 50 Hz (±1 Hz bandwidth) was added to eliminate electric power line interference. The filtered signal is then full-wave rectified to convert it to absolute values. To further smooth the rectified signal, a fourth-order zero-phase low-pass Butterworth filter with a cutoff frequency of 8 Hz was used. A 100 ms moving window was used to calculate the root mean square (RMS) value (Hermens et al., 2000). The RMS value obtained during each exercise bout was normalized by dividing the maximum RMS value recorded during the MVIC trial of the corresponding muscle to quantify the relative activation level of the muscle. This normalization process yielded MVIC% values, which represent relative muscle activation levels during exercise. All signal processing steps were implemented in MATLAB R2023a (MathWorks, United States of America), and the filter coefficients were generated using the filtfilt function to ensure zero-phase filtering (Kim et al., 2018). This comprehensive and carefully designed signal processing pipeline ensures that the EMG signals are accurately captured and analyzed, thereby providing a robust basis for the subsequent interpretation of the results.

### 2.4 Bench press test with different tempos and loads

To facilitate subsequent statistical analysis, the four modes in this study were coded as follows: a) 60% (X/0/X/0), b) 60% (2/0/2/0), c) 80% (X/0/X/0), and d) 80% (2/0/2/0). The order of tempos for the bench press was randomly determined by lot. Participants performed four sets of bench press training to exhaustion according to the different tempo sequences, with the EMG changes of the target muscles recorded throughout. The rest period between each set was 5 min. During the experiment, a metronome (60 bpm (Beats per minute), i.e., one beat per second) was used to regulate the participants’ bench press tempo, ensuring that each repetition strictly followed the metronome’s rhythm. The standard of bench press movement was consistent with that in the 1RM test, and a dedicated person supervised and protected the participants during the experiment. In addition, the Vmaxpro sensor was used to record the instantaneous velocity of the barbell at a sampling rate of 200 Hz, documenting the participants’ movement velocity and power under specific loads to evaluate the degree of exercise fatigue. Measurements were taken before and after training. Moreover, blood lactate was measured before and after the bench press test. Following the protocol of Mang et al. (2022), a handheld lactate analyzer (Lactate Plus, NOVA Biomedical the maximum velocity tempo, MA) was used for blood lactate measurement. Researchers disinfected the earlobe with an alcohol swab, then pricked the earlobe to draw blood (Lynge et al., 2024). The first drop of blood was wiped away with gauze, and the second drop was collected for measurement. Two measurements were taken before and after exercise, and the average of the repeated readings was recorded. The specific test indicators are shown in Table 2.

**TABLE 2:** Specific indicators included in the bench press test with different tempos and loads.

Experimental indicator	Experimental equipment	Impact on fatigue	Index calculation method
MVIC%, MDF	Surface Electromyography	Lower MVIC% indicates lower muscle activation and more pronounced fatigue; Lower MDF values indicate more pronounced fatigue (González-Izal et al., 2010).	MVIC% = $\frac{\text{RMS}}{{\text{RMS}}_{\text{MVIC}}}$ ×100 MDF = *f*_median_ Where $\sum_{f=0}^{MDF}P\left(f\right)=\frac{1}{2}\sum_{f=0}^{\infty }P\left(f\right)$
Power and Velocity	Vmaxpro Sensor	Lower MV, PV, MP, and PP indicate more pronounced muscle fatigue. Higher PV loss rate indicates higher fatigue (Krzysztofik et al., 2020).	MV = $\frac{\Delta s}{\Delta t}$ PV = Vmax PV loss Rate = $\frac{{PV}_{set1}-{PV}_{setn}}{{PV}_{set1}}$ MP = $\frac{1}{T}\int_{0}^{T}F\left(t\right)v\left(t\right)dt$ PP = max_t_ [F(t)v(t)] TUT = $\sum_{i=1}^{n}t_{ecc,i}+t_{iso,i}+t_{con,i}$
Blood Lactate	Lactate Analyzer	Higher blood lactate levels indicate more pronounced fatigue (Okawara et al., 2022).	The concentration of fingertip blood samples was determined directly using a lactate analyzer

### 2.5 Statistical analysis

Data from the experiment are presented as mean ± standard deviation. ImageJ and GraphPad Prism 8.0 were used for data visualization (ImageJ was used for image analysis of EMG signals, and GraphPad Prism 8.0 was used for generating figures), and SPSS 22.0 was employed for statistical analysis. After homogeneity of variance testing, t-tests were used for pairwise comparisons in univariate analysis, and two-way repeated-measures ANOVA (set × mode) was used. Mauchly’s test assessed sphericity; violations were corrected with Greenhouse–Geisser. Bonferroni *post hoc* tests were applied.

## 3 Results

### 3.1 Effects of bench press training with different loads and tempos on EMG indicators

#### 3.1.1 Effects of bench press training with different tempos and loads on MVIC%

Intraclass comparisons revealed that in the 60% (x/0/x/0) group, the MVIC% values in the third set (P = 0.009) and fourth set (P = 0.000) were significantly higher than those in the first set, and the fourth set was significantly higher than the second set (P = 0.004). No other significant differences were observed within the groups. Between groups with the same load but different rhythms, the MVIC% value in the fourth set of the 60% (x/0/x/0) group was significantly higher than that of the 60% (2/0/2/0) group (P = 0.012). Between groups with the same rhythm but different loads, the MVIC% value in the fourth set of the 80% (2/0/2/0) group was significantly higher than that of the 60% (2/0/2/0) group (P = 0.002). The specific results are shown in Table 3.

**TABLE 3:** Changes in MVIC% of the right pectoralis major muscle during bench press training with different tempos and loads (*n* = 10).

Muscle group	Exercise mode	Bench press set			
		Set 1	Set 2	Set 3	Set 4
Right anterior deltoid	60% (X/0/X/0)	41.2 ± 10.7	51.4 ± 9.32	55.8 ± 6.97*	66.7 ± 4.79*△
	60% (2/0/2/0)	43.6 ± 7.76	48.4 ± 11.1	49.8 ± 5.14	52.9 ± 8.77
	80% (X/0/X/0)	50.8 ± 9.26	53 ± 7.68	56.6 ± 6.18	60.4 ± 4.54
	80% (2/0/2/0)	54.4 ± 8.84	57.8 ± 6.1	62.7 ± 8.62	68.4 ± 8.91§
Between-group effect	F = 9.77, P = <0.001, η^2^ = 0.170				
Time effect	F = 29.51, P = <0.001, η^2^ = 0.257				
Time × group interaction	F = 2.00, P = <0.050, η^2^ = 0.052				

* denotes within-group comparison, P < 0.05; △ denotes between-group comparison at the same load but different cadences, △<0.05; § denotes between-group comparison at the same cadence but different loads, §<0.05.

Intraclass comparisons revealed that in the 80% (x/0/x/0) group, the MVIC% value in the fourth set was significantly higher than that in the first set (P = 0.029), with no other significant differences observed within the groups. Between groups with the same load but different rhythms, the MVIC% value of the right middle deltoid muscle in the 60% (x/0/x/0) group during the fourth set of bench presses was significantly higher than that in the 60% (2/0/2/0) group (p = 0.026). Similarly, the MVIC% value of the right middle deltoid muscle in the 80% (x/0/x/0) group during the fourth set was significantly higher than that in the 80% (2/0/2/0) group (p = 0.033). The specific results are shown in Table 4.

**TABLE 4:** Changes in MVIC% of the right anterior deltoid muscle during bench press training with different tempos and loads (*n* = 10).

Muscle group	Exercise mode	Bench press set			
		Set 1	Set 2	Set 3	Set 4
Right anterior deltoid	60% (X/0/X/0)	41.3 ± 5.16	47.6 ± 15.1	52.8 ± 14.6	57.5 ± 11.6△
	60% (2/0/2/0)	30.7 ± 7.89	34 ± 8.03	37.4 ± 10.3	33.8 ± 9.54
	80% (X/0/X/0)	48.3 ± 6.17	56 ± 12.3	62.1 ± 17.6	65.4 ± 17.8*△
	80% (2/0/2/0)	36.3 ± 11.6	38.8 ± 16.4	45.1 ± 12.2	42.2 ± 11.1
Between-group effect	F = 12.3, P = <0.001, η^2^ = 0.341				
Time effect	F = 12.28, P = <0.001, η^2^ = 0.078				
Time × group interaction	F = 1.12, P = <0.001, η^2^ = 0.021				

* denotes within-group comparison, P < 0.05; △ denotes between-group comparison at the same load but different cadences, △<0.05; § denotes between-group comparison at the same cadence but different loads, §<0.05.

Intraclass comparisons revealed that in the 80% (x/0/x/0) group, the MVIC% value of the right triceps brachii during the third set of bench presses was significantly higher than that in the first set (p = 0.018). Between groups with the same load but different rhythms, the MVIC% value of the right triceps brachii in the 60% (x/0/x/0) group during the second set (p = 0.015), third set (p = 0.003), and fourth set (p = 0.029) of bench presses was significantly higher than that in the 60% (2/0/2/0) group. Additionally, the MVIC% value of the right triceps brachii in the 80% (x/0/x/0) group during the third set of bench presses was significantly higher than that in the 80% (2/0/2/0) group (p = 0.041). The specific results are shown in Table 5.

**TABLE 5:** Changes in MVIC% of the right triceps brachii muscle during bench press training with different tempos and loads (*n* = 10).

Muscle group	Exercise mode	Bench press set	Muscle group	Exercise mode	Bench press set
		Set 1	Set 2	Set 3	Set 4
Right triceps brachii	60% (X/0/X/0)	34.6 ± 11.6	43.5 ± 7.27^△^	46.5 ± 6.7^△^	47 ± 8.74^△^
	60% (2/0/2/0)	19.7 ± 10.4	22.4 ± 10.8	24.3 ± 12.1	24.7 ± 14.1
	80% (X/0/X/0)	38.6 ± 11.1	48 ± 13.4	52.6 ± 14^*△^	50.4 ± 15.7
	80% (2/0/2/0)	30.2 ± 3.92	36.6 ± 11.5	34.3 ± 6.19	37.2 ± 9.08
Between-group effect	F = 23.8, P = <0.001, η^2^ = 0.418				
Time effect	F = 7.698, P = <0.001, η^2^ = 0.063				
Time × group interaction	F = 0.568, P = 0.795, η^2^ = 0.014				

* denotes within-group comparison, P < 0.05; △ denotes between-group comparison at the same load but different cadences, △<0.05; § denotes between-group comparison at the same cadence but different loads, §<0.05.

#### 3.1.2 Analysis of the effects of bench press training with different tempos and loads on Median Frequency (MDF)

Intraclass comparisons revealed that in the 60% (x/0/x/0) group, the MDF value in the first set was significantly higher than that in the fourth set (P = 0.003). In the 80% (x/0/x/0) group, the MDF values in the first set (P = 0.000), second set (P = 0.012), and third set (P = 0.047) were significantly higher than those in the fourth set. Comparisons between groups with the same rhythm but different loads showed that in the 60% (x/0/x/0) group, the MDF values in the first set (P = 0.012), third set (P = 0.002), and fourth set (P = 0.006) were significantly higher than those in the 80% (x/0/x/0) group. Additionally, in the 60% (2/0/2/0) group, the MDF values in the first set (P = 0.025), second set (P = 0.041), third set (P = 0.000), and fourth set (P = 0.004) were significantly higher than those in the 80% (2/0/2/0) group. The specific results are shown in Table 6.

**TABLE 6:** Changes in MDF of the right pectoralis major muscle during bench press training with different tempos and loads (*n* = 10).

Muscle group	Exercise mode			Bench press set	
		Set 1	Set 2	Set 3	Set 4
Right pectoralis major	60% (X/0/X/0)	4.60 ± 0.4*§	4.39 ± 0.32	4.31 ± 0.20§	3.89 ± 0.39^§^
	60% (2/0/2/0)	4.38 ± 0.15§	4.28 ± 0.30§	4.20 ± 0.29§	4.03 ± 0.44§
	80% (X/0/X/0)	4.04 ± 0.09*	3.91 ± 0.42*	3.72 ± 0.35*	3.32 ± 0.18
	80% (2/0/2/0)	3.85 ± 0.36	3.70 ± 0.20	3.58 ± 0.14	3.37 ± 0.10
Between-group effect	F = 42.0, P = <0.001, η^2^ = 0.399				
Time effect	F = 30.67, P = <0.001, η^2^ = 0.213				
Time × group interaction	F = 1.08, P = 0.381, η^2^ = 0.023				

* denotes within-group comparison, P < 0.05; △ denotes between-group comparison at the same load but different cadences, △<0.05; § denotes between-group comparison at the same cadence but different loads, §<0.05.

Intraclass comparisons revealed that in the 60% (x/0/x/0) group, the MDF value in the first set was significantly higher than that in the fourth set (P = 0.035). Similarly, in the 80% (x/0/x/0) group, the MDF value in the first set was significantly higher than that in the fourth set (P = 0.028). Comparisons between groups with the same rhythm but different loads showed that in the first set, the MDF value of the 60% (2/0/2/0) group was significantly higher than that of the 80% (2/0/2/0) group (P = 0.034). The specific results are shown in Table 7.

**TABLE 7:** Changes in MDF of the right anterior deltoid muscle during bench press training with different tempos and loads (*n* = 10).

Muscle group	Exercise mode	Bench press set			
		Set 1	Set 2	Set 3	Set 4
Right anterior deltoid	60% (X/0/X/0)	4.35 ± 0.40*	4.24 ± 0.37	4.12 ± 0.4	3.75 ± 0.37
	60% (2/0/2/0)	4.30 ± 0.30	4.17 ± 0.33	4.12 ± 0.38	4.02 ± 0.33
	80% (X/0/X/0)	3.97 ± 0.327*§	3.84 ± 0.58	3.55 ± 0.67	3.36 ± 0.37
	80% (2/0/2/0)	3.72 ± 0.24	3.75 ± 0.21	3.68 ± 0.90	3.58 ± 0.20
Between-group effect	F = 18.2, P = <0.001, η^2^ = 0.246				
Time effect	F = 9.203, P = <0.001, η^2^ = 0.114				
Time × group interaction	F = 0.868, P = 0.552, η^2^ = 0.032				

* denotes within-group comparison, P < 0.05; △ denotes between-group comparison at the same load but different cadences, △<0.05; § denotes between-group comparison at the same cadence but different loads, §<0.05.

Intraclass comparisons revealed that in the 60% (x/0/x/0) group, the MDF value in the first set was significantly higher than that in the fourth set (P = 0.035). Similarly, in the 80% (x/0/x/0) group, the MDF value in the first set was significantly higher than that in the fourth set (P = 0.028). Comparisons between groups with the same rhythm but different loads showed that in the first set, the MDF value of the 60% (2/0/2/0) group was significantly higher than that of the 80% (2/0/2/0) group (P = 0.034). The specific results are shown in Table 8.

**TABLE 8:** Changes in MDF of the right triceps brachii muscle during bench press training with different tempos and loads (*n* = 10).

Muscle group	Exercise mode	Bench press set			
		Set 1	Set 2	Set 3	Set 4
Right triceps brachii	60% (X/0/X/0)	4.82 ± 0.28*§	4.41 ± 0.32	4.25 ± 0.36	3.97 ± 0.42§
	60% (2/0/2/0)	4.45 ± 0.61	4.34 ± 0.62	4.23 ± 0.61	4.02 ± 0.58
	80% (X/0/X/0)	3.80 ± 0.80*	3.63 ± 0.61	3.38 ± 0.44	3.01 ± 0.49
	80% (2/0/2/0)	3.92 ± 0.36	3.82 ± 0.44	3.60 ± 0.66	3.76 ± 0.22
Between-group effect	F = 28.2, P = <0.001, η^2^ = 0.317				
Time effect	F = 8.655, P = <0.001, η^2^ = 0.101				
Time × group interaction	F = 0.843, P = 0.572, η^2^ = 0.029				

* denotes within-group comparison, P < 0.05; △ denotes between-group comparison at the same load but different cadences, △<0.05; § denotes between-group comparison at the same cadence but different loads, §<0.05.

### 3.2 Analysis of the effects of different rhythms and loads on power output during bench press training

#### 3.2.1 Analysis of the effects of different rhythms and loads on MV, PV, and peak velocity loss rate during bench press training

Intraclass comparisons revealed that in the 60% (x/0/x/0) group, the MV value in the first set was significantly higher than that in the fourth set (P = 0.047). Similarly, in the 60% (2/0/2/0) group, the MV value in the first set was significantly higher than that in the fourth set (P = 0.022). In the 80% (x/0/x/0) group, the MV value in the first set was significantly higher than that in the third (P = 0.001) and fourth sets (P = 0.000). Comparisons between groups with the same load but different rhythms showed that the MV values in the 60% (x/0/x/0) group were significantly higher than those in the 60% (2/0/2/0) group across all sets (P < 0.001). In the 80% (x/0/x/0) group, the MV values in the first to third sets were significantly higher than those in the 80% (2/0/2/0) group (P < 0.001). Comparisons between groups with the same rhythm but different loads revealed that the MV values in the second to fourth sets of the 60% (x/0/x/0) group were significantly higher than those in the 80% (x/0/x/0) group (P < 0.001). Additionally, the MV values in the 60% (x/0/x/0) group were significantly higher than those in the 80% (x/0/x/0) group across all sets of bench presses (P < 0.001). The specific results are shown in Table 9.

**TABLE 9:** Changes in MV during bench press training under different rhythms and loads (*n* = 10).

Muscle group	Exercise mode	Bench press set			
		Set 1	Set 2	Set 3	Set 4
Mean velocity (MV)	60% (x/0/x/0)	0.46 ± 0.04*△§	0.44 ± 0.03△§	0.42 ± 0.04△§	0.39 ± 0.05△§
	60% (2/0/2/0)	0.31 ± 0.06*	0.29 ± 0.05	0.26 ± 0.04	0.23 ± 0.02
	80% (x/0/x/0)	0.38 ± 0.04*△	0.33 ± 0.04△	0.30 ± 0.04△	0.26 ± 0.05
	80% (2/0/2/0)	0.25 ± 0.03	0.23 ± 0.03	0.19 ± 0.04	0.20 ± 0.03
Between-group effect	F = 120, P = <0.001, η^2^ = 0.700				
Time effect	F = 35.22, P = <0.001, η^2^ = 0.107				
Time × group interaction	F = 1.52, P = 0.154, η^2^ = 0.014				

* denotes within-group comparison, P < 0.05; △ denotes between-group comparison at the same load but different cadences, △<0.05; § denotes between-group comparison at the same cadence but different loads, §<0.05.

Intraclass comparisons revealed that in the 60% (x/0/x/0) group, the PV value in the first set was significantly higher than that in the fourth set (P = 0.013); in the 80% (x/0/x/0) group, the PV value in the first set was significantly higher than that in the third (P = 0.000) and fourth sets (P = 0.000). Comparisons between groups with the same load but different rhythms showed that the PV values in the 60% (x/0/x/0) group were significantly higher than those in the 60% (2/0/2/0) group across all sets (P < 0.001); in the 80% (x/0/x/0) group, the PV values in the first to third sets were significantly higher than those in the 80% (2/0/2/0) group (P = 0.000, P = 0.000, P = 0.034, respectively). Comparisons between groups with the same rhythm but different loads revealed that the PV values in the second to fourth sets of the 60% (x/0/x/0) group were significantly higher than those in the 80% (x/0/x/0) group (P < 0.001). The specific results are shown in Table 10.

**TABLE 10:** Changes in PV during bench press training under different rhythms and loads (*n* = 10).

Muscle group	Exercise mode	Bench press set			
		Set 1	Set 2	Set 3	Set 4
Peak velocity (PV)	60% (x/0/x/0)	0.64 ± 0.02*△§	0.60 ± 0.04△§	0.58 ± 0.06△§	0.54 ± 0.10△§
	60% (2/0/2/0)	0.37 ± 0.04	0.34 ± 0.04	0.33 ± 0.03	0.31 ± 0.03
	80% (x/0/x/0)	0.31 ± 0.08*△	0.29 ± 0.06△	0.28 ± 0.08△	0.27 ± 0.03
	80% (2/0/2/0)	0.31 ± 0.04	0.29 ± 0.04	0.28 ± 0.03	0.27 ± 0.04
Between-group effect	F = 109, P = <0.001, η^2^ = 0.792				
Time effect	F = 28.96, P = <0.001, η^2^ = 0.049				
Time × group interaction	F = 2.18, P = 0.043, η^2^ = 0.011			

* denotes within-group comparison, P < 0.05; △ denotes between-group comparison at the same load but different cadences, △<0.05; § denotes between-group comparison at the same cadence but different loads, §<0.05.

Intraclass comparisons revealed that in the 80% (2/0/2/0) group, the velocity loss rate in the third set was significantly higher than that in the first set (P = 0.001). Comparisons between groups with the same load but different rhythms showed that the loss rate in the first set of the 60% (x/0/x/0) group was significantly higher than that of the 60% (2/0/2/0) group (P = 0.037); the loss rate in the first set (P = 0.001) and fourth set (P = 0.04) of the 80% (x/0/x/0) group was significantly higher than that of the 80% (2/0/2/0) group. Comparisons between groups with the same rhythm but different loads revealed that the loss rate in the third set of the 80% (2/0/2/0) group was significantly higher than that of the 60% (2/0/2/0) group (P = 0.038). The specific results are shown in Table 11.

**TABLE 11:** Changes in peak velocity loss rate during bench press training under different rhythms and loads (*n* = 10).

Muscle group	Exercise mode	Bench press set			
		Set 1	Set 2	Set 3	Set 4
Mean velocity loss rate (%)	60% (x/0/x/0)	15.2 ± 5.55△	17.1 ± 5.39	16.7 ± 3.05	17.7 ± 2.34
	60% (2/0/2/0)	7.59 ± 1.35	11 ± 3.61	12.8 ± 3.44	12.8 ± 4.09
	80% (x/0/x/0)	20 ± 4.79△	17.7 ± 7.49	17.4 ± 4.52	21.5 ± 5.04△
	80% (2/0/2/0)	10.3 ± 4.03	13.9 ± 4.23	20 ± 4.89*§	14.5 ± 3.9
Between-group effect	F = 17, P = <0.001, η^2^ = 0.277				
Time effect	F = 6.24, P = <0.001, η^2^ = 0.064				
Time × group interaction	F = 3.16, P = 0.005, η^2^ = 0.097				

* denotes within-group comparison, P < 0.05; △ denotes between-group comparison at the same load but different cadences, △<0.05; § denotes between-group comparison at the same cadence but different loads, §<0.05.

#### 3.2.2 Analysis of the effects of different rhythms and loads on MP and peak power (PP) during bench press training

Comparisons between groups with the same load but different rhythms revealed that the MP values in the 60% (x/0/x/0) group were significantly higher than those in the 60% (2/0/2/0) group in the first (P < 0.001), second (P < 0.001), and fourth sets (P < 0.001). In the 80% (x/0/x/0) group, the MP values were significantly higher than those in the 80% (2/0/2/0) group across all sets (P < 0.001 or P = 0.04). Comparisons between groups with the same rhythm but different loads showed that the MP values in the 60% (x/0/x/0) group were significantly higher than those in the 80% (x/0/x/0) group in the first (P = 0.006) and fourth sets (P = 0.017). The MP values in the 60% (2/0/2/0) group were significantly higher than those in the 80% (2/0/2/0) group in the first (P = 0.047), third (P = 0.000), and fourth sets (P = 0.017). The specific results are shown in Table 12.

**TABLE 12:** Changes in MP during bench press training under different rhythms and loads**.**

Muscle group	Exercise mode		Bench press set		
		Set 1	Set 2	Set 3	Set 4
Mean power (MP)	60% (x/0/x/0)	362 ± 54.5△§	350 ± 38.6△	341 ± 36.7	332 ± 48.1△§
	60% (2/0/2/0)	241 ± 29.3§	223 ± 35.6§	270 ± 65.6	196 ± 18
	80% (x/0/x/0)	282 ± 33.7△	306 ± 50.2△	307 ± 37.9△	264 ± 45.3△
	80% (2/0/2/0)	173 ± 32.6	163 ± 13.6	147 ± 20	160 ± 17.7
Between-group effect	F = 81.3, P = <0.001, η^2^ = 0.740				
Time effect	F = 709, P = <0.001, η^2^ = 0.020				
Time × group interaction	F = 3.42, P = 0.001, η^2^ = 0.029				

* denotes within-group comparison, P < 0.05; △ denotes between-group comparison at the same load but different cadences, △<0.05; § denotes between-group comparison at the same cadence but different loads, §<0.05.

Intraclass comparisons revealed that in the 60% (x/0/x/0) group, the PP value in the second set was significantly higher than that in the fourth set (P = 0.033). In the 80% (x/0/x/0) group, the PP values in the third (P = 0.008) and fourth sets (P = 0.042) were significantly lower than that in the first set. In the 80% (2/0/2/0) group, the PP values in the second (P = 0.005) and third sets (P = 0.035) were significantly lower than that in the first set. Comparisons between groups with the same load but different rhythms showed that the PP values in the 60% (x/0/x/0) group were significantly higher than those in the 60% (2/0/2/0) group across all sets (P < 0.001). In the 80% (x/0/x/0) group, the PP values were significantly higher than those in the 80% (2/0/2/0) group across all sets (P = 0.01, P = 0.000, P = 0.002, P = 0.04). Comparisons between groups with the same rhythm but different loads revealed that the PP value in the third set of the 60% (x/0/x/0) group was significantly higher than that in the 80% (x/0/x/0) group (P = 0.002). The PP value in the first set of the 80% (2/0/2/0) group was significantly higher than that in the 60% (2/0/2/0) group (P = 0.022). The specific results are shown in Table 13.

**TABLE 13:** Changes in PP during bench press training under different rhythms and loads**.**

Muscle group	Exercise mode	Bench press set			
		Set 1	Set 2	Set 3	Set 4
Peak power (PP)	60% (x/0/x/0)	469 ± 76.5△	460 ± 46.5*△	447 ± 60.1△§	411 ± 73.8△
	60% (2/0/2/0)	229 ± 20.7	225 ± 21.5	220 ± 25.9	217 ± 32.5
	80% (x/0/x/0)	419 ± 41.3△	397 ± 50△	357 ± 41.9*△	357 ± 38.9*△
	80% (2/0/2/0)	321 ± 41.7§	271 ± 37.1*	266 ± 24.9*	263 ± 30.4
Between-group effect	F = 72.8, P = <0.001, η^2^ = 0.772				
Time effect	F = 21.45, P = <0.001, η^2^ = 0.033				
Time × group interaction	F = 2.83, P = 0.020, η^2^ = 0.013				

* denotes within-group comparison, P < 0.05; △ denotes between-group comparison at the same load but different cadences, △<0.05; § denotes between-group comparison at the same cadence but different loads, §<0.05.

#### 3.2.3 Analysis of the effects of different rhythms and loads on TUT during bench press training

Intraclass comparisons revealed that in the 60% (x/0/x/0) group, the TUT value in the first set was significantly higher than that in the fourth set (P = 0.001). Similarly, in the 60% (2/0/2/0) group, the TUT value in the first set was significantly higher than that in the fourth set (P = 0.006). In the 80% (2/0/2/0) group, the TUT value in the first set was significantly higher than that in the fourth set (P = 0.000). Comparisons between groups with the same load but different rhythms showed that the TUT values in the 60% (2/0/2/0) group were significantly higher than those in the 60% (x/0/x/0) group across all sets (P < 0.001). In the 80% (x/0/x/0) group, the TUT values were significantly higher than those in the 80% (2/0/2/0) group across all sets (P < 0.001). Comparisons between groups with the same rhythm but different loads revealed that the TUT values in the 60% (x/0/x/0) group were significantly higher than those in the 80% (x/0/x/0) group across all sets (P = 0.003, P = 0.007, P = 0.045, P = 0.009). The TUT values in the 60% (2/0/2/0) group were significantly higher than those in the 80% (2/0/2/0) group across all sets (P < 0.001). The specific results are shown in Table 14.

**TABLE 14:** Changes in TUT during bench press training under different rhythms and loads**.**

Muscle group	Exercise mode	Bench press set			
		Set 1	Set 2	Set 3	Set 4
TUT	60% (x/0/x/0)	53.4 ± 8.17*§	48.2 ± 5.87§	42.4 ± 5.49§	40.4 ± 5.25§
	60% (2/0/2/0)	81.5 ± 6.63*△§	77.7 ± 6.78△§	71.1 ± 6.94△§	69.9 ± 8.1△§
	80% (x/0/x/0)	37.5 ± 6.48	34.3 ± 2.73	30.3 ± 5.03	27.9 ± 3.87
	80% (2/0/2/0)	64.7 ± 7.95*△	57.1 ± 9.89△	52.1 ± 9.15△	49.3 ± 6.83△
Between-group effect	F = 172, P = <0.001, η^2^ = 0.784				
Time effect	F = 33.467, P = <0.001, η^2^ = 0.076				
Time × group interaction	F = 0.391, P = 0.922, η^2^ = 0.003				

* denotes within-group comparison, P < 0.05; △ denotes between-group comparison at the same load but different cadences, △<0.05; § denotes between-group comparison at the same cadence but different loads, §<0.05.

#### 3.2.4 Analysis of the effects of different rhythms and loads on blood lactate during bench press training

The experimental results showed that the blood lactate value after exhaustive bench press testing in the 60% (x/0/x/0) group was significantly higher than that in the 60% (2/0/2/0) group (p = 0.000). The blood lactate values after exhaustive bench press testing in the 80% (x/0/x/0) group (p = 0.000) and the 80% (2/0/2/0) group (P = 0.002) were significantly higher than that in the 60% (2/0/2/0) group. The blood lactate value after exhaustive bench press testing in the 80% (x/0/x/0) group was significantly higher than that in the 80% (2/0/2/0) group (p = 0.014). The specific results are shown in Table 15.

**TABLE 15:** Changes in blood lactate before and after bench press training under different rhythms and loads**.**

	Exercise mode			
	60% (x/0/x/0)	60% (2/0/2/0)	80% (x/0/x/0)	80% (2/0/2/0)
Pre-test	2.43 ± 0.15	2.33 ± 0.22	2.43 ± 0.11	2.29 ± 0.30
Post-test	7.48 ± 0.21*	6.4 ± 0.60	7.92 ± 0.42*	7.23 ± 0.30*
Between-group effect	F = 34.5, P = <0.001, η^2^ = 0.014			
Time effect	F = 3441.6, P = <0.001, η^2^ = 0.960			
Time × group interaction	F = 12.6, P = <0.001, η^2^ = 0.011			

* denotes between-group comparison at post-test, P < 0.05.

## 4 Discussion

This study aimed to systematically investigate the effects of bench-press protocols combining different loading conditions (moderate load at 60% 1RM vs. high load at 80% 1RM) and movement tempos (maximal velocity X/0/X/0 vs. medium tempo 2/0/2/0) on exercise-induced fatigue in bodybuilders. The results demonstrated that both load and tempo substantially modulated EMG activity, kinetic performance, and the onset of fatigue. Maximal-velocity tempo (X/0/X/0) elicited higher muscle activation (MVIC %) in the later stages of training—particularly in the fourth set—most prominently in the pectoralis major at 60% 1RM, and in the anterior deltoid and triceps brachii across loading conditions. Conversely, high-load (80% 1RM) training, irrespective of tempo, induced a more pronounced decline in MDF of the target muscles, indicating greater local muscular fatigue. Kinetically, MV, PV, MP, and PP were all significantly higher under maximal-velocity tempo than under medium tempo, although peak-velocity loss rates were occasionally greater. Metabolically, blood-lactate accumulation was markedly higher under maximal-velocity tempo, reaching its maximum at 80% 1RM. Overall, within the same loading condition, maximal-velocity tempo (X/0/X/0) yielded higher MVIC % values than medium tempo (2/0/2/0), suggesting elevated muscle activation and increased neural drive requirements. Meanwhile, high-load (80% 1RM) training, especially when performed at specific tempos, was associated with greater blood-lactate accumulation, reflecting enhanced metabolic stress and peripheral exercise-induced fatigue.

The training efficacy of the bench press is governed by multiple factors, among which training load and movement tempo are two critical modulatory variables. Distinct load–tempo combinations generate differentiated training stimuli, thereby exerting profound effects on performance, fatigue magnitude, and eventual training adaptations. Load represents one of the most central variables in resistance training, directly influencing both mechanical performance and fatigue development. In general, as load increases, the number of repetitions that can be performed decreases, movement velocity declines, and power output may vary within specific ranges. Hunter et al. compared bench-press exercise performed at intensities ranging from 20% to 80% 1RM and observed that energetic economy deteriorated with increasing intensity, i.e., more energy was required to perform a given amount of work at higher loads (Hunter et al., 1988). Blood-lactate concentrations after trials at 60% 1RM were significantly higher than after 30% 1RM, yet no differences were detected between 60% 1RM and two trials at 70% 1RM—an outcome likely attributable to variations in total work or repetition volume across the protocols. A subsequent study that employed maximum-repetition bench-press schemes at 60% 1RM and 90% 1RM reported markedly higher post-exercise blood-lactate levels following the 60% 1RM protocol, presumably because of the greater number of repetitions and total volume completed (Maté-Muñoz et al., 2025). Collectively, these findings indicate that load not only affects the mechanical output of individual repetitions but also modulates metabolite accumulation and fatigue via its influence on repetition number and total work. High loads (≥80% 1RM) are typically employed to maximize strength and impose greater neural demands, often resulting in central fatigue, whereas moderate loads (60%–80% 1RM) are commonly used for hypertrophy or strength-endurance development and may elicit more pronounced metabolite accumulation and peripheral fatigue. In addition to load, tempo strategies—such as rapid concentric actions, slow eccentric actions, or deliberate pauses between eccentric–concentric transitions—alter TUT, muscle-activation patterns, and metabolic stress. When total work is matched, slower tempos (e.g., 6 s per repetition) produce higher blood-lactate concentrations and greater perceived exertion (RPE) than faster tempos (e.g., 2 s per repetition) (Arazi et al., 2013). This is presumably because prolonged muscle contraction under slower tempos restricts local blood flow, slows metabolite clearance, and accelerates accumulation of by-products such as lactate, H^+^, and inorganic phosphate, thereby hastening fatigue [Muscle fatigue: general understanding and treatment]. Another study comparing different tempos (4-1-4-1, 2-1-2-1, 1-1-1-1, and maximal-velocity concentric) during bench-press exercise revealed that slower tempos (e.g., 4-1-4-1) resulted in longer exercise duration and higher oxygen consumption, indirectly reflecting greater metabolic stress and potential fatigue accumulation (Buitrago et al., 2012). Fast-tempo training—particularly concentric actions performed with maximal intent—is associated with power development. Although time under tension per contraction is brief, moderate loads allow multiple repetitions to be completed in a short timeframe, yielding substantial cumulative metabolic stress. Lacerda et al. compared two Smith-machine bench-press protocols matched for total TUT (6 reps × 6 s vs. 12 reps × 3 s, both at 60% 1RM) and observed significantly greater EMG activity and higher blood-lactate concentrations in the protocol employing more repetitions with shorter repetition duration (12 × 3 s) (Lacerda et al., 2016). These data indicate that, even when total TUT is identical, faster tempos (shorter concentric/eccentric times or fewer pauses) can induce greater metabolic stress and muscular activation. However, Pallarés et al. (2014) reported that bench-press and squat movements with a 2-s pause between eccentric and concentric phases (absence of stretch-shortening cycle, SSC) exhibited lower variability in individual load–velocity profiles and smaller errors in %1RM estimation than non-paused movements. This indirectly underscores that tempo (inclusion or absence of pauses) influences movement execution and the stability of mechanical output. Nevertheless, fast-tempo training—characterized by high power output and rapid energy turnover—may rapidly deplete phosphagen stores and, via rapid muscle lengthening–shortening, impose greater mechanical stress, thereby increasing the risk of muscle damage.

It should be emphasized that load and tempo do not influence bench-press performance and fatigue in isolation; rather, tempo modulation under different load magnitudes may evoke divergent physiological and mechanical responses, while a given tempo strategy can elicit distinct performance and fatigue profiles across loads. Load and tempo—manifested through repetition number and inter-set rest—jointly determine training density and metabolic stress (Sánchez-Moreno et al., 2023). The present experimental design directly examined the interaction between two loads (60% and 80% 1RM) and two tempos (X/0/X/0, maximal velocity; 2/0/2/0, medium tempo), thereby offering further insight into this intricate relationship. The MVIC % trajectories observed herein indicate that maximal-velocity tempo (X/0/X/0) elevated MVIC % during the latter sets—particularly under moderate load (60% 1RM) for the pectoralis major and across loads for the anterior deltoid and triceps brachii. This suggests that, when rapid concentric actions are required, the nervous system must intensify neural drive to recruit additional motor units, especially high-threshold fast-twitch fibres, to overcome resistance and sustain performance. Conversely, although 80% 1RM protocols induced greater fatigue, they simultaneously produced higher muscle activation, corroborating previous findings. Haun et al. (2017) reported significantly greater sEMG amplitudes in lower-limb muscles during 80% 1RM knee-extension exercise than during 30% 1RM, despite greater integrated EMG in the light-load, higher-repetition condition. Under heavy loads, muscles must generate greater force to overcome higher resistance, elevating sEMG amplitude from the outset; as fatigue progresses, further increases in EMG activity likely reflect enhanced neural drive and/or additional motor-unit recruitment to offset force decline (Tsoukos and Bogdanis, 2023). In contrast, medium tempo (2/0/2/0) controlled both concentric and eccentric phases but elicited lower or more gradual increases in muscle activation in later sets. Moreover, under medium tempo, pectoralis major MVIC % in the fourth set was significantly higher at 80% 1RM than at 60% 1RM, indicating that high load *per se* imposes greater activation demands when tempo is fixed. These observations partially align with Lacerda et al. [Variations in Repetition Duration and Repetition Numbers Influence Muscular Activation and Blood Lactate Response in Protocols Equalized by Time Under Tension], who demonstrated that Smith-machine bench-press protocols entailing more repetitions with shorter repetition durations (i.e., a relatively “faster” tempo) evoked higher EMG activity. MDF reduction is conventionally associated with fatigue and reflects slowed fibre-conduction velocity, metabolite accumulation, and diminished fast-twitch fibre contribution. The present findings reveal that high-load (80% 1RM) training, irrespective of tempo, consistently produced more pronounced MDF declines in the target muscles (pectoralis major, anterior deltoid, triceps brachii), denoting greater localised fatigue. This is attributable to the greater fast-twitch fibre solicitation and the concomitant accelerated energy depletion and metabolite build-up elicited by heavy loads. Although maximal-velocity tempo yielded higher muscle activation, its influence on MDF appeared to be predominantly load-dependent. Collectively, distinct load–tempo combinations differentially modulate neural drive strategies, motor-unit recruitment quantity and phenotype (fast vs. slow fibres), and the intramuscular metabolic milieu, thereby shaping muscle activation and fatigue development in a load- and tempo-specific manner.

Alterations in kinetic variables provide a direct reflection of movement quality and the progression of neuromuscular fatigue during bench-press training. The present results demonstrate that velocity- and power-related indices (MV, PV, MP, PP) exhibited a consistent pattern: a maximum velocity tempo (X/0/X/0) combined with a low load (60% 1RM) elevated initial velocity and power, yet these parameters declined markedly as fatigue accumulated; conversely, a medium tempo (2/0/2/0) paired with a high load (80% 1RM) induced greater velocity loss and shortened total TUT. TUT displayed an inverse relationship with velocity and power, being jointly determined by tempo and load: longer TUT was observed under slower tempos and lower loads. Notably, despite the maximal-velocity tempo (X/0/X/0) producing superior initial velocity, force, and power outputs, it also elicited a higher velocity-loss rate under certain conditions; specifically, peak-velocity loss from set 1 to set 4 was significantly greater in the 80% (X/0/X/0) condition than in the 80% (2/0/2/0) condition. This suggests that maximal-intent training, particularly at higher loads, delivers potent neuromuscular stimulation yet accelerates the onset of neuromuscular fatigue. Collectively, the data indicate that low-load protocols help preserve force-generating capacity and attenuate fatigue accumulation, whereas high-load protocols, while initially promoting high force output, expedite neuromuscular exhaustion. Fast-tempo conditions enable rapid attainment of peak force and power, but velocity and force efficiency decline more steeply as fatigue develops. These findings align with previous work by Varela-Olalla et al. (2024), who reported that low-load bench-press exercise performed to failure, despite permitting faster initial bar velocities, results in greater total mechanical work and metabolic stress because of the higher repetition volume, ultimately leading to more pronounced decrements in force and velocity in later sets. Such losses in power and velocity are likely attributable to transient reductions in central drive, impaired neuromuscular junction transmission, and rapid depletion of fast-twitch fibre energy stores (Sergeeva et al., 2024).

Blood lactate is a well-established marker of anaerobic glycolytic activity, and its accumulation is closely linked to exercise intensity and metabolic stress. The present findings demonstrate that, at 60% 1RM, blood-lactate concentrations were significantly higher when the lift was performed with a maximal-velocity tempo (X/0/X/0) than with a medium tempo (2/0/2/0). This observation is partially consistent with the report of Lacerda et al. [Variations in Repetition Duration and Repetition Numbers Influence Muscular Activation and Blood Lactate Response in Protocols Equalized by Time Under Tension], who noted that, when total time-under-tension was matched, shorter repetition durations (i.e., faster tempos) combined with higher repetition counts elicited greater lactate accumulation. Under the 60% 1RM condition employed here, the X/0/X/0 tempo likely allowed participants to complete more repetitions before failure or to perform more work per unit time, thereby increasing glycolytic flux and lactate production. When load was increased to 80% 1RM, blood-lactate levels in the maximal-velocity (X/0/X/0) group remained significantly higher than in the moderate-tempo (2/0/2/0) group. This pattern mirrors the 60% 1RM results and further supports the metabolic-stress-inducing advantage of a maximum velocity tempo. Notably, lactate concentrations in the 80% 1RM (2/0/2/0) condition were also significantly elevated compared with 60% 1RM (2/0/2/0), indicating that load itself exerts a potent lactate-promoting effect. These observations align with the findings of Nitzsche et al. (2018), who reported that increasing load augmented lactate accumulation during blood-flow-restriction exercise. Persistent lactate accumulation alters the metabolic milieu—via H^+^ and lactate build-up—which can impair enzymatic activity, Ca^2+^ release and binding, and actomyosin cross-bridge cycling, thereby precipitating fatigue (Sánchez-Moreno et al., 2023). When the current results are compared with those of Vargas-Molina et al. (2020), careful attention must be paid to tempo definitions and experimental design. Salvador et al. observed that, with total TUT fixed at 60 s, a high-repetition, slower-tempo protocol (HRP: 20 reps, 2-s eccentric/1-s concentric) produced higher lactate than a moderate-repetition, faster-tempo protocol (MRP: 10 reps, 4-s eccentric/2-s concentric). The “slower” HRP and “faster” MRP classifications differ from the present maximal-velocity X/0/X/0 and controlled 2/0/2/0 tempos. In the current study, X/0/X/0 emphasizes maximal concentric explosiveness; if this tempo enabled a relatively large number of repetitions to be completed at high velocity, power output and metabolic demand per unit time would be elevated, accelerating lactate accumulation. Nevertheless, the present results clearly indicate that, at any given load, a maximal concentric-velocity tempo elicits higher blood-lactate levels than a controlled medium tempo. The 80% 1RM (X/0/X/0) group displayed the highest lactate concentration across all conditions, underscoring the maximal metabolic challenge imposed by combining high load with maximal velocity. The underlying physiological mechanisms likely involve rapid phosphagen depletion due to maximal mechanical and physiological loading, prompt activation of glycolysis, and rapid accumulation of lactate and H^+^. Concurrently, high-frequency neural drive may precipitate neuromuscular-junction failure and central-drive decline, while elevated shear forces within muscle and connective tissues contribute to peripheral fatigue (Wilk et al., 2021b).

The present study has the following limitations: First, the sample size was relatively small, with only 10 male bodybuilders included. This may limit the generalizability of the study findings to females, non-athletes, or individuals engaged in other sports. Future research should expand the sample size and include participants of different genders and training levels to further validate the universality and applicability of these findings. Second, the study assessed fatigue solely based on EMG, kinetics, and blood lactate levels, without incorporating multidimensional indicators such as subjective perception of fatigue (e.g., the Rating of Perceived Exertion, RPE scale), neurofunctional measures (e.g., reaction time), or hormonal levels (e.g., cortisol, testosterone). This singular approach to indicator selection may affect the in-depth elucidation of fatigue mechanisms, such as the interaction between central and peripheral fatigue. Future research should consider integrating more multidimensional indicators to provide a more comprehensive understanding of the impact of different training variables on fatigue.

Core contribution: The primary contribution of this study lies in its first systematic quantification, via a rigorous two-factor crossover design, of the interactive effects of load (60% vs. 80% 1RM) and tempo (maximal velocity X/0/X/0 vs. medium speed 2/0/2/0) on multifaceted peripheral fatigue responses during bench press training. The results demonstrate that while the high-load and fast-tempo combination (80% 1RM + X/0/X/0) elicits the most potent muscle activation and metabolic stress (e.g., significantly elevated blood lactate), it concurrently induces the most pronounced declines in neuromuscular efficiency, movement velocity, and power output, indicating it generates acute, multidimensional peripheral fatigue. In contrast, the high-load, medium-tempo protocol (80% 1RM + 2/0/2/0) results in less acute fatigue despite a longer time under tension (TUT). This key finding underscores that the interaction between load and tempo—not either variable alone—is the critical determinant of the nature and magnitude of the fatigue response, providing an empirical basis for precisely managing fatigue and optimizing training prescriptions in resistance training.

## 5 Conclusion

The present study reached the following conclusions: Bench press training at 80% load (X/0/X/0) significantly induced peripheral fatigue responses, characterized by higher muscle activation (significant increase in MVIC% values), more pronounced peripheral fatigue responses (significant decrease in MDF values), and greater metabolic stress (significant elevation in blood lactate levels). In contrast, the combination of medium tempo (2/0/2/0) and high load can prolong the TUT, but the degree of fatigue is relatively lower. Additionally, training at 60% load (X/0/X/0) can maintain higher MV and PP, but the rate of velocity loss significantly increases with the accumulation of sets, indicating a faster accumulation of fatigue. Under 80% load, the power output of the fast-tempo group decreased more markedly, suggesting that the combination of high load and maximum velocity tempo has a more significant acute fatigue effect on explosive training.

## 6 Additional notes

In your research, X/0/X/0 represents both centrifugal and centripetal stages completed at maximum velocity tempo without pause, emphasizing explosive force and neural drive in rhythm control. Its characteristics are high initial velocity and power, but decrease faster with the training process, especially under high load (80% 1RM), which can cause greater metabolic pressure (significant increase in lactate), decrease in electromyographic frequency, and accumulation of peripheral fatigue. Therefore, this rhythm is suitable for short-term high-intensity stimulation to enhance explosive power and neural adaptation, but at the same time, attention needs to be paid to fatigue management and recovery strategies.

In this study, 2/0/2/0 denotes a controlled tempo with 2 s for eccentric and concentric phases and no pauses, emphasizing stability and prolonged TUT. Compared with X/0/X/0, it induced lower acute fatigue at the same relative loads, while still providing sufficient mechanical tension, especially under 80% 1RM. This tempo is therefore more suitable for enhancing muscular endurance, technical control, and hypertrophic stimulus with relatively manageable fatigue levels.

## Data Availability

The raw data supporting the conclusions of this article will be made available by the authors, without undue reservation.
